# Pregnancy outcomes and genetic analysis for fetal ventriculomegaly

**DOI:** 10.3389/fgene.2023.1186660

**Published:** 2023-09-15

**Authors:** Huimin Tao, Lin Zhang, Fangfang Tan, Yu Han, Xuezhen Wang, Jiebin Wu, Jingfang Zhai

**Affiliations:** ^1^ Xuzhou Central Hospital, Xuzhou Clinical College of Xuzhou Medical University, Xuzhou, China; ^2^ Key Laboratory of Brain Diseases Bioinformation of Xuzhou Medical University, Xuzhou, China; ^3^ Department of Prenatal Diagnosis Medical Center, Xuzhou Central Hospital, Xuzhou, China; ^4^ School of Information and Control Engineering, China University of Mining and Technology, Xuzhou, Jiangsu, China

**Keywords:** fetus, ventriculomegaly, genetic analysis, multidisciplinary evaluation, pregnancy outcomes

## Abstract

**Introduction:** Fetal ventriculomegaly (VM) is associated with neurodevelopmental disorders, partly caused by genetic factor.

**Methods:** To systematically investigate the genetic etiology of fetal VM and related pregnancy outcomes in different subgroups: IVM (isolated VM) and NIVM (non-isolated VM); unilateral and bilateral VM; mild, moderate, and severe VM, a retrospective study including 131 fetuses with VM was carried out from April 2017 to August 2022.

**Results:** 82 cases underwent amniocentesis or cordocentesis, of whom 8 cases (9.8%) were found chromosomal abnormalities by karyotyping. Meanwhile, additional 8 cases (15.7%) with copy number variations (CNVs) were detected by copy number variation sequencing (CNV-seq). The detection rate (DR) of chromosomal abnormalities was higher in NIVM, bilateral VM and severe VM groups. And CNVs frequently occurred in NIVM, bilateral VM and moderate VM groups. In the NIVM group, the incidence of chromosomal aberrations and CNVs in multiple system anomalies (19.0%, 35.7%) was higher than that in single system anomalies (10.0%, 21.1%). After dynamic ultrasound follow-up, 124 cases were available in our cohort. 12 cases were further found other structural abnormalities, and lateral ventricular width was found increased in 8 cases and decreased in 15 cases. Meanwhile, 82 cases underwent fetal brain MRI, 10 cases of brain lesions and 11 cases of progression were additionally identified. With the involvement of a multidisciplinary team, 45 cases opted for termination of pregnancy (TOP) and 79 cases were delivered with live births. One infant death and one with developmental retardation were finally found during postnatal follow-ups.

**Discussion:** CNV-seq combined with karyotyping could effectively improve the diagnostic rate in fetuses with VM. Meanwhile, dynamic ultrasound screening and multidisciplinary evaluation are also essential for assessing the possible outcomes of fetuses with VM.

## Introduction

Fetal lateral ventricular width, usually 7.6 ± 0.6 mm from 15 to 40 weeks of gestation, is a routine examination parameter in prenatal ultrasound screening. Fetal ventriculomegaly (VM) is defined as the width of one or both sides of the lateral ventricles ≥10 mm according to ultrasound diagnostic criteria (Yagel and Valsky, 2021), with a prevalence of 0.3‰–1.5‰ (Myrianthopoulos, 1977). Fetal VM can be classified into different subtypes: mild (10–12 mm), moderate (13–15 mm), severe (>15 mm) VM (Griffiths et al., 2010); unilateral and bilateral VM; isolated VM (IVM) and non-isolated VM (NIVM) indicating plus other multi-system abnormalities. It has been reported that 7.9% fetuses with mild IVM may have neurodevelopmental retardation, and the prevalence of adverse outcomes may increase to 38%–90% in fetuses with severe IVM or hydrocephalus (Pagani et al., 2014; Letouzey et al., 2017; Thorup et al., 2019). Therefore, it is pivotal for clinicians to predict the possible prognosis of the affected fetuses.

The outcomes of fetal VM can be influenced by a variety of factors such as chromosomal abnormality, viral infection and progression *in utero*. Hence, it makes prenatal counseling more challenging for clinicians. The Society for Maternal-Fetal Medicine (SMFM) recommends that clinicians should perform a comprehensive evaluation for fetuses with VM, including detailed fetal structures, invasive prenatal diagnosis for genetic etiology, and a workup for fetal infections (Fox et al., 2018). Compared with traditional karyotyping, copy number variation sequencing (CNV-seq) based on next-generation sequencing has outstanding advantages of high resolution, high throughput and whole genome coverage. In recent years, it has been widely applied in the field of prenatal diagnosis (Zhao and Fu, 2019). In this study, we focused on analyzing the value of genetic analysis, dynamic ultrasound screening, and multidisciplinary evaluation for the purpose of improving perinatal management in fetuses with different types of VM.

## Materials and methods

### Study subjects

**FIGURE 1:**
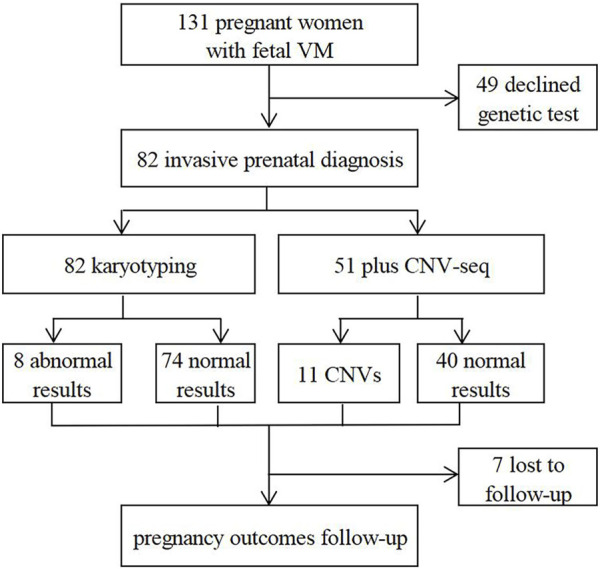
Flow chart of clinical management procedures.

**FIGURE 2:**
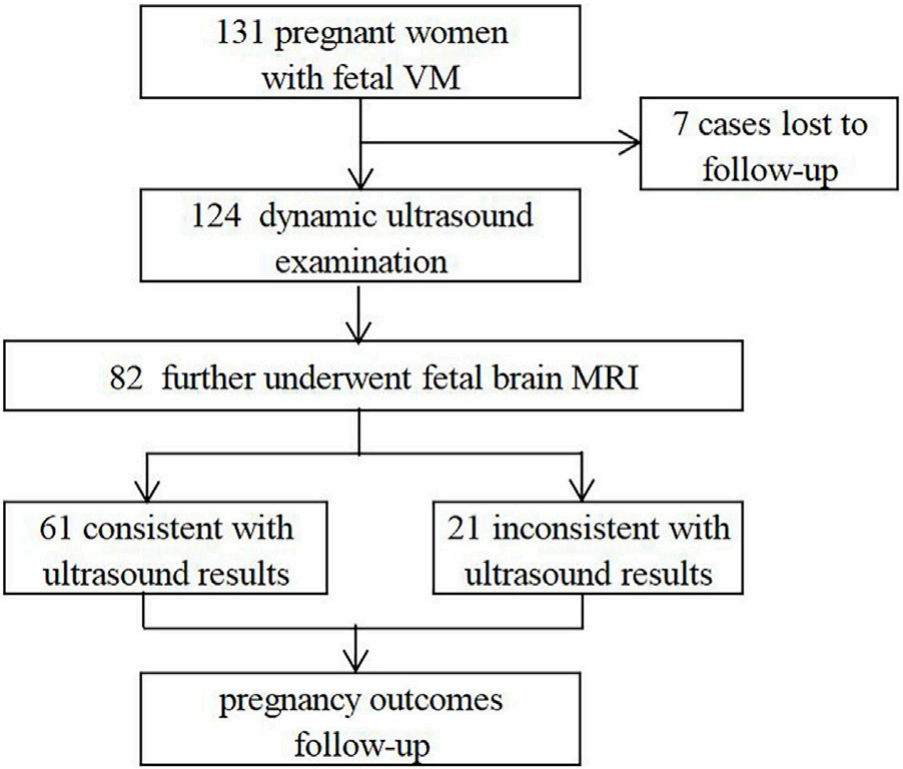
Flow chart of imaging diagnosis pathway.

In this retrospective study, 131 fetuses diagnosed with VM by prenatal ultrasound were enrolled at the prenatal diagnosis center of Xuzhou Central Hospital from April 2017 to August 2022. Patients with underlying diseases or high-risk factors were ruled out, such as hypertension, immune diseases, trauma, twin or multiple pregnancy, *etc.* All pregnant women received prenatal counseling and provided written informed consents for further examinations. 82 cases underwent invasive prenatal diagnosis for karyotyping, and 51 cases simultaneously underwent CNV-seq (Figure 1). 124 cases were further evaluated by dynamic ultrasound examination and 82 cases further underwent fetal brain MRI (Figure 2). Our study has been approved by the ethics committee of Xuzhou Central Hospital (XZXY-LJ-2017- 0626-003).

### Ultrasound examination and MRI

Prenatal ultrasound scanning was performed by ultrasound physicians using a 2.5∼5 MHz transabdominal transducer and color doppler ultrasound instrument (GE Voluson E8, American) according to the guidelines of the International Society of Ultrasound in Obstetrics and Gynecology (ISUOG) (Salomon et al., 2022). Fetal VM was diagnosed as the width of lateral ventricles ≥10 mm at the level of the choroid plexus perpendicular to the ventricular cavity (Yagel and Valsky, 2021).

Fetal MRI was performed by experienced operators using a 1.5T scanner (Infineon, Philips Medical Systems). The transverse diameter of the trigones of both lateral ventricles was measured and any additional central nervous system (CNS) abnormalities should also be carefully examined (Fox et al., 2018). The results were then compared with ultrasound findings.

### Karyotyping and CNV-seq

51 amniotic fluid samples and 31 cord blood samples were collected by ultrasound-guided amniocentesis or cordocentesis. These samples were then subjected to the following procedures: *in situ* cell culture, harvesting, G-banding, and result judgment. All karyotyping reports were interpreted in line with the International System for Human Cytogenetic Nomenclature at the level of 300–400 bands (ISCN 2016).

Fetal genomic DNA extraction and library construction were conducted following laboratory standard operating procedures. The following massive whole-genome sequencing (0.1x) in parallel was carried out using the NextSeqCN500 platform (Illumina, United States). 50 ng fetal DNA from each prenatal sample was used for CNV-seq. The sequencing data were analyzed by GISTIC 2.0 with reference to the human reference genome sequence version GRCh37/hg19, the standards of the American College of Medical Genetics (ACMG) (Richards et al., 2015; Riggs et al., 2020), and related databases such as Database of Genomic Variants (DGV). For the cases with abnormal CNVs, parental peripheral blood samples were also suggested to further verify whether it was inherited or *de novo*.

### Follow-up of pregnancy outcomes

An experienced multidisciplinary team (MDT) from many departments, including ultrasound, magnetic resonance imaging (MRI), prenatal diagnosis and neurology, worked together to assess the possible prognosis of fetuses with VM. Pregnancy outcomes were recorded by postpartum telephone and electronic medical record system. Neonates and infants were followed up from 6 months to 2 years after birth and comprehensively assessed in terms of neurodevelopmental outcomes by experienced pediatricians.

### Statistical analyses

The data were analyzed by SPSS software (version 26.0, IBM, Armonk, NY, United States). Continuous variables were expressed as mean ± standard deviation (SD), and independent sample t-test was used for comparison between groups. Enumeration data were expressed as frequency, and chi-square test or Fisher’s exact test was used to compare the rates between groups (*p* < 0.05 was regarded as statistically significant).

## Results

### Basic characteristics of study subjects

131 fetuses with VM were included in our study, including 50 cases with IVM and 81 cases with NIVM. The basic characteristics were listed in Table 1. The mean maternal age (MA) was 28.76 ± 4.91 years, 20 (15.3%) were in advanced age (≥35 years old); the mean gestational age (GA) was 26.75 ± 4.31 weeks; nulliparas accounted for 45.0%. Five pregnant women had histories of adverse pregnancy: 2 induced labor due to hydrocephalus, 2 fetal cardiac anomalies, and 1 fetal cervical hygroma. No obvious statistical differences were observed in MA, GA and parity (*p* > 0.05). The percentages of mild, moderate and severe VM were 74.0%, 19.1% and 6.9%, respectively. There were 58.8% fetuses with unilateral VM and 41.2% with bilateral VM. Except 7 cases (5.3%) lost to follow-up, 79 cases (60.3%) were delivered and 45 cases (34.4%) opted for termination of pregnancy (TOP). Significant differences were found between IVM and NIVM groups in the degree of VM (mild: 88.0% vs. 65.4%; moderate: 10.0% vs. 24.7%; severe: 2.0% vs. 9.9%), lateral of VM (unilateral: 72.0% vs. 50.6%; bilateral: 28.0% vs. 49.4%) and pregnancy outcomes (delivery: 88.0% vs. 43.2%; TOP: 8.0% vs. 50.6%; Lost to follow-up: 4.0% vs. 6.2%) (*p* < 0.05).

**TABLE 1:** Basic characteristics of study subjects.

Characteristics	Total	IVM	NIVM	χ^2^/t	*P*
MA (years)					
<35	111 (84.7)	44 (88.0)	67 (82.7)	0.667	0.414
≥35	20 (15.3)	6 (12.0)	14 (17.3)		
Mean MA	28.76 ± 4.91	28.92 ± 4.07	28.65 ± 5.39	0.325	0.746
Mean GA (weeks)	26.75 ± 4.31	27.13 ± 3.71	26.51 ± 4.65	0.842	0.401
Parity					
Nulliparous	59 (45.0)	21 (42.0)	38 (46.9)	0.302	0.583
Parous	72 (55.0)	29 (58.0)	43 (53.1)		
Degrees of VM					
Mild	97 (74.0)	44 (88.0)	53 (65.4)		
Moderate	25 (19.1)	5 (10.0)	20 (24.7)	8.415	0.015
Severe	9 (6.9)	1 (2.0)	8 (9.9)		
Lateral of VM					
Unilateral	77 (58.8)	36 (72.0)	41 (50.6)		
Bilateral	54 (41.2)	14 (28.0)	40 (49.4)	5.834	0.016
Pregnancy outcomes					
Delivery	79 (60.3)	44 (88.0)	35 (43.2)		
TOP	45 (34.4)	4 (8.0)	41 (50.6)	26.904	<0.001
Lost to follow-up	7 (5.3)	2 (4.0)	5 (6.2)		
Total	131 (100)	50 (38.2)	81 (61.8)		

^a^ MA: maternal age, GA: gestational age, VM: ventriculomegaly, TOP: termination of pregnancy, IVM: isolated ventriculomegaly, NIVM: non-isolated ventriculomegaly.

### Abnormal karyotypes in different subtypes of fetal VM

Chromosomal abnormalities were found in 8 cases (9.8%) by karyotyping, including trisomy 21 (*n* = 3), mos46,XX [43]/46,XY [57], mos47,XXY [21]/46,XY [79], 46,XY,inv (9) (p12q21.1), 46,XX,del (5) (q14), and 46,XX,del (1) (p36) (Tables 2, 3). Although no significant differences were observed in the subtypes (*p* > 0.05): IVM and NIVM groups (3.2% vs. 13.7%); unilateral and bilateral VM groups (6.0% vs. 15.6%); mild, moderate and severe VM groups (9.2% vs. 9.1% vs. 16.7%), we found that abnormal results mostly occurred in NIVM, bilateral VM and severe VM.

**TABLE 2:** Chromosomal abnormalities in fetal VM detected by karyotyping.

Karyotyping	Total	IVM	NIVM	Unilateral	Bilateral	Mild	Moderate	Severe
Total	82	31	51	50	32	65	11	6
Abnormal	8	1	7	3	5	6	1	1
Normal	74	30	44	47	27	59	10	5
Positive rate	9.8%	3.2%	13.7%	6.0%	15.6%	9.2%	9.1%	16.7%
χ^2^		2.414		2.053		0.351		
*P*		0.120		0.152		0.839		

^a^ IVM: isolated ventriculomegaly, NIVM: non-isolated ventriculomegaly.

**TABLE 3:** Abnormal chromosomes, CNVs and pregnancy outcomes in 16 fetuses with VM.

Case	GA	Karyotyping	CNVs	Size (Mb)	Type	US findings	Outcomes
1	33	mos46,XX [43]/46,XY [57]	-	-	-	Severe VM; Absence of corpus callosum	TOP
2	29 + 3	mos47,XXY [21]/46,XY [79]	-	-	-	Mild VM; Posterior fossa widened	Delivery
3	25 + 6	47,XX,+21	-	-	-	Mild VM; Ventricular bright spot; Thickened nuchal fold	TOP
4	18 + 5	47,XY,+21	-	-	-	Mild VM; Echogenic bowel	TOP
5	24 + 3	47,XY,+21	dup (21) (q11.1q22.3)	33.83	P	Moderate VM; Absence of nasal bone; Ventricular septal defect	TOP
6	25 + 6	46,XY,inv (9) (p12q21.1)	N	N	N	Mild VM	Delivery
7	29 + 4	46,XX,del (5) (q14)	del (5) (q14.3q15)	7.94	P	Mild VM; Arachnoid cyst	TOP
8	24 + 6	46,XX,del (1) (p36)	del (1) (p36.33p36.22)	8.70	P	Mild VM; Posterior fossa widened; Aortic stenosis	TOP
9	23 + 2	46,XY	del (1) (p36.32p36.21)	2.80	VOUS (maternal)	Mild VM; Arachnoid cyst; Double renal pelvis	Delivery
10	24	46,XX	del (16) (p13.11)	0.78	VOUS	Mild VM	TOP
11	23 + 5	46,XX	del (16) (p11.2)	0.48	LP (paternal)	Moderate VM; Strephenopodia	TOP
12	26 + 2	46,XX	dup(X) (p22.12)	1.10	VOUS	Mild VM	Delivery
13	21 + 3	46,XX	del(X) (p22.13p22.12)	1.00	P	Moderate VM; Dandy-Walker malformation; Absence of cavum septi pellucid	TOP
14	27 + 1	46,XY	del (16) (p11.2)	0.42	LP	Mild VM; Fetal growth restriction	TOP
15	25 + 1	46,XY	dup (6) (q23.3)	1.40	VOUS	Moderate VM; Absence of cavum septi pellucid; Absence of corpus callosum	TOP
16	29	46,XY	dup (11) (q25)	0.98	LP	Mild VM; Posterior fossa widened	Delivery

^a^ undone; N: normal; P: pathogenic; LP: likely pathogenic; VOUS: variants of uncertain significance; TOP: termination of pregnancy.

### CNVs in different subtypes of fetal VM

11 cases (21.6%) with CNVs were detected by CNV-seq in the 51 subjects, including pathogenic CNVs (P) (*n* = 4; dup (21) (q11.1q22.3), del (5) (q14.3q15) associated with Bosch-Boonstra-Schaaf optic atrophy syndrome (BBSOAS), del (1) (p36.33p36.22) associated with 1p36 microdeletion syndrome, del(X) (p22.13p22.12) associated with developmental and epileptic encephalopathy 2 and retinoschisis 1), likely pathogenic CNVs (LP) (*n* = 3; del (16) (p11.2) associated with 16p11.2 deletion syndrome (*n* = 2), dup (11) (q25)), and variants of uncertain significance (VOUS) (*n* = 4; del (1) (p36.32p36.21), del (16) (p13.11), dup(X) (p22.12), dup (6) (q23.3)) (Table 3; Table 4). 3 cases were consistent with karyotyping results, while the remaining 8 (15.7%) were additionally revealed by CNV-seq, indicating that CNV-seq could effectively make up for the deficiency of karyotyping. Furthermore, the DR of CNVs was higher in fetuses with NIVM than those with IVM (27.3% vs 11.1%), bilateral VM than unilateral VM (22.7% vs 20.7%), and moderate VM than mild VM (40.0% vs 18.4%). However, no statistical differences were shown in the above three groups (*p* > 0.05).

**TABLE 4:** CNVs in fetal VM detected by CNV-seq.

CNV-seq	Total	IVM	NIVM	Unilateral	Bilateral	Mild	Moderate	Severe
Total	51	18	33	29	22	38	10	3
CNVs	11	2	9	6	5	7	4	0
Normal	40	16	24	23	17	31	6	3
Positive rate	21.60%	11.1%	27.3%	20.7%	22.7%	18.4%	40.0%	0.0%
χ^2^		1.798		0.031		3.415		
*P*		0.180		0.861		0.181		

### Correlation between chromosomal aberrations and indicators for NIVM

In the NIVM group, 51 cases underwent the molecular genetic test, including 30 single system anomalies and 21 multiple system anomalies (Table 5). Although the DR of both chromosomal abnormalities and CNVs were higher in multi-system anomalies (19.0%, 35.7%) than in single system anomalies (10.0%, 21.1%), no significant differences were observed between the two groups (*p* > 0.05). It suggested that chromosomal abnormalities are the ones that increase the risk of NIVM. What’s more, cardiovascular system abnormalities were mostly found in multi-system anomalies. 25.0% (3/12) abnormal karyotypes and 25.0% (2/8) CNVs were revealed when combined with cardiovascular abnormalities.

**TABLE 5:** Chromosomal aberrations in NIVM.

US findings	Karyotyping (n = 51)					CNV-seq (n = 33)				
	Total	Nor	Abn	χ^2^	*P*	Total	Nor	Abn	χ^2^	*P*
Single system	30	27	3	0.854	0.355	19	15	4	0.874	0.350
≥2 systems	21	17	4			14	9	5		
CNS	4	3	1			4	2	2		
CVS	12	9	3			8	6	2		
Digestive system	3	2	1			1	1	0		
Urinary system	2	2	0			1	0	1		
Skeletal system	2	1	1			2	1	1		
Others	4	3	1			3	1	2		

^a^ CNS: central nervous system; CVS: cardiovascular system; Nor: normal; Abn: abnormal; Others including thickened nuchal fold, fetal growth restriction, strephenopodia, polyhydramnios.

### Follow-up outcomes

After dynamic ultrasound screening, 124 fetuses with VM were available in the cohort (Figure 2). 12 cases were further found other structural abnormalities, and lateral ventricular width was also found increased in 8 cases and decreased in 15 cases. Meanwhile, 82 cases underwent fetal brain MRI, and 21 were inconsistent with ultrasound findings, including 10 brain lesions with poor prognosis (agenesis of the corpus callosum (ACC) (n = 8), Dandy-Walker malformation (DWM) (*n* = 1), cerebral cortical growth retardation (*n* = 1)) and 11 cases of lateral ventricular width progression. It suggested that fetal brain MRI can improve the diagnosis of additional CNS anomalies and monitor the degree of VM.

**FIGURE 3:**
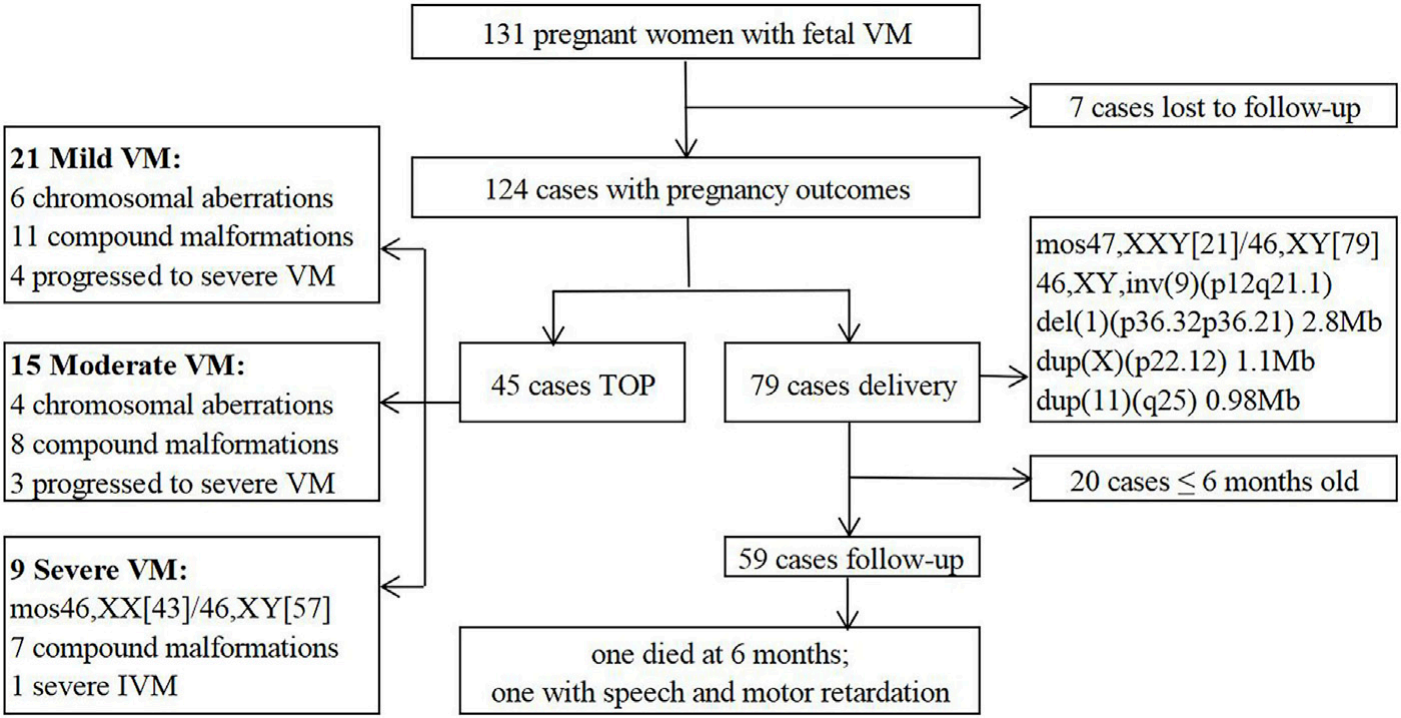
Flow chart of follow-up outcomes.

Finally, 79 (63.7%) cases continued the pregnancy and 45 (36.3%) opted for TOP (Figure 3), most of whom followed the recommendations of MDT. The latter 45 cases included mild VM (*n* = 21), moderate VM (*n* = 15) and severe VM (*n* = 9). In the mild VM group, 6 cases were terminated due to chromosomal aberrations (Case 3, 4, 7, 8, 10, 14); 11 cases complicated with other malformations (ACC (*n* = 3), DWM (*n* = 2), cerebral cortical growth retardation (*n* = 2), holoprosencephaly (HPE) (*n* = 1), cerebellum dysplasia (*n* = 1), myelomeningocele (*n* = 1), bilateral polycystic kidneys and oligohydramnios (n = 1)); 4 cases progressed to severe VM. In the moderate VM group, 4 cases were terminated due to chromosomal aberrations (Case 5, 11, 13, 15); 8 cases complicated with other anomalies (ACC (n = 2), HPE (n = 1), HPE and cleft lip and palate (n = 1), choroid plexus papilloma (n = 1), polyhydramnios and bilateral renal pelvis separation (n = 1), cerebral cortical growth retardation (n = 1), atrial and ventricular septal defect (n = 1)); 3 cases progressed to severe VM. Moreover, 9 cases were terminated in the severe VM group, including chromosomal XX/XY mosaicism (Case 1); isolated severe VM (n = 1); 7 cases complicated with other abnormalities (posterior fossa widened (n = 2), ACC (n = 2), septo-optic dysplasia, HPE and ventricular septal defect (n = 1), spina bifida (n = 1), aqueductal stenosis and encephalomalacia (n = 1)).

79 cases continued the pregnancy, including 5 premature babies and 74 full-term babies (51 boys and 28 girls). 5 cases were found chromosomal aberrations (Case 2, 6, 9, 12, 16). The prognosis of the 5 fetuses was predicted to be relatively good by MDT based on imaging and genetic information. Postnatal follow-ups were performed in 59 subjects from 6 months to 2 years after birth. One infant without genetic test died at 6 months due to progressive VM and ACC. One infant with mild unilateral VM and normal karyotype was found speech and motor retardation at 2 years old. No abnormalities were found in the remaining 57 children.

## Discussion

Fetal VM may increase the risk of neurodevelopmental abnormalities after birth, such as autism, schizophrenia, and epilepsy (Chang et al., 2020; Lok et al., 2021; Chen et al., 2022). Our study highlighted the importance of genetic etiology in fetal VM, and indicated that karyotyping together with CNV-seq can effectively improve the detection rate of fetuses with VM.

Previous studies have shown that chromosomal abnormalities occurred in 5%–20% of fetuses with IVM, and pathogenic CNVs are also common causes accounting for about 4.20% (Pagani et al., 2014; Chang et al., 2020; Wang et al., 2020). In our study, 9.8% cases with VM were found chromosomal abnormalities by karyotyping, meanwhile, additional 15.7% CNVs were detected by CNV-seq (Table 2; Table 4). Some are recognized as microdeletion or microduplication syndromes, including BBSOAS, 1p36 microdeletion syndrome, and 16p11.2 deletion syndrome. The DR of chromosomal aberrations in fetuses with VM was higher by CNV-seq than by traditional karyotyping, regardless of single or multi-system anomalies. It suggested that CNV-seq can effectively improve the DR of fetuses with VM over karyotyping (Wang et al., 2021). It is not hard to see that fetuses with NIVM, bilateral VM or moderate-to-severe VM have a higher risk of chromosomal abnormalities or CNVs. Therefore, more attention should be paid to the above fetuses, to effectively avoid birth defects with genetic factors. In fetuses with NIVM, the DR of chromosomal aberrations in multi-system anomalies was higher than that in single system anomalies (Table 5), which is consistent with the findings of Chang (Chang et al., 2020). It suggested that chromosomal abnormalities are the ones that increase the risk of NIVM. Thus, when fetal VM combined with multiple malformations, especially cardiovascular system, we should be more aware of the possibility of chromosomal aberrations. From our point of view, karyotyping combined with CNV-seq should be recommended to rule out genetic abnormalities, especially for fetuses with NIVM, bilateral VM or moderate-to-severe VM.

It is often difficult to reveal fetal phenotypes at a certain point in time by prenatal ultrasound due to the limitations of the fetal position, gestational age, and amniotic fluid volume. In our study, during dynamic ultrasound screening, additional structural abnormalities in 12 patients and an increase or regression of lateral ventricular width in 23 patients were revealed. Therefore, dynamic ultrasound follow-up is of great significance to avoid under/overdiagnosis and assess the possible prognosis to facilitate better management for the affected fetuses. In addition, 10 cases of fetal brain lesions with poor prognosis were detected by MRI but missed by ultrasound, and 11 cases with lateral ventricular width progression were also detected. Therefore, fetal brain MRI can act as an effective complementary tool to confirm the diagnosis and reveal additional CNS anomalies.

Based on the above strategies, multidisciplinary consultation should be recommended for couples on whether to continue or terminate the pregnancy, especially in the late second or third trimesters, which might comprehensively provide better perinatal management for fetuses with VM. Regarding pregnancy outcomes, 79 cases continued the pregnancy with live births including 5 cases diagnosed with genetic etiology (a low proportion of sex chromosomes mosaicism (20% 47, XXY and 80% 46,XY), inversion of chromosome 9, microdeletion in 1p36.33p36.22 inherited from the mother, microduplications in Xp22.11 and 11q25). From our experience, the involvement of MDT, as well as parental persistence, contributed to the continuation of pregnancy. Postnatal follow-ups from 6 months to 2 years showed 1 neonatal death at 6 months, and 1 speech and motor retardation at 2 years old. A large population-based study explained that even in apparent IVM, there was a significant risk of postnatal additional structural anomalies and neonatal death (Hannon et al., 2012). Hence, even for mild, isolated VM, we cannot leave out the possibility of poor results. It is pivotal for MDT to carefully evaluate fetuses with different types of VM both prenatally and postpartum. In our data, TOP was chosen in all cases with severe VM. When VM is the only abnormal finding, the parents make decisions mostly based on the severity of VM, as the poor outcomes increase with the degree of VM aggravated. Whether the degree of VM can act as an independent risk factor to judge fetal prognosis needs to be further explored through larger sample sizes. It is also the responsibility of MDT to carefully make a recommendation for TOP after comprehensive consideration.

There are still some limitations in our study that need to be improved. First, fetal brain MRI has been proven as a useful tool to identify additional CNS anomalies, such as ACC associated with poor outcomes (Wang et al., 2020), but not all cases in our study received the examination. Secondly, tests for pathogenic microorganisms such as cytomegalovirus and toxoplasmosis should be performed to rule out infectious factors (SMFM et al., 2020). Last but not least, the follow-up time and sample size were not sufficient to find neurodevelopmental abnormalities in the children. Longer follow-ups and more cases will be enrolled in the future to explore the outcomes of fetal VM.

In short, CNV-seq could effectively improve the diagnostic rate of fetal VM, over karyotyping. Meanwhile, dynamic ultrasound screening and multidisciplinary evaluation are also essential for better management of fetuses with VM.

## Data Availability

The datasets presented in this study can be found in online repositories. The names of the repository/repositories and accession number(s) can be found below: https://www.ncbi.nlm.nih.gov/, PRJNA944381.
